# Case report: Simultaneous occurrence of primary pulmonary lymphoma and opportunistic infections in a patient with chronic myeloid leukemia

**DOI:** 10.3389/fonc.2022.1031500

**Published:** 2022-10-18

**Authors:** Yazhen Bi, Saran Feng, Jinyu Shang, Qian Liu, Yan Wang

**Affiliations:** ^1^ Department of Hematology, Shandong Provincial Qianfoshan Hospital, Shandong University, Jinan, China; ^2^ Department of Hematology, The First Affiliated Hospital of Shandong First Medical University & Shandong Provincial Qianfoshan Hospital, Jinan, China; ^3^ Graduate School, Shandong First Medical University, Jinan, China

**Keywords:** chronic myeloid leukemia, primary pulmonary lymphoma, opportunistic infections, antibiotic therapy, biopsy, pathology

## Abstract

**Background:**

The occurrence of primary pulmonary lymphoma (PPL) as a secondary malignancy in patients diagnosed with chronic myeloid leukemia (CML) is extremely rare. As the clinical manifestations are atypical, most patients with PPL tend to be misdiagnosed with pneumonia. When the radiographic features of PPL and pulmonary infection overlap, clinicians can be confused about the diagnosis. Here, we report the first case of coexistence of PPL and opportunistic infections in a patient with CML in chronic phase (CML-CP).

**Case presentation:**

A 55-year-old woman presented with three weeks of hemorrhage of the oral mucosa at the Department of Hematology. After undergoing various examinations, she was diagnosed with CML-CP and was started on imatinib (400 mg/daily). Due to sudden respiratory distress, the patient was admitted to the respiratory intensive care unit 11 months later. Chest computed tomography (CT) revealed ground-glass opacities, patchy shadows, and multiple nodules in both lungs and enlarged mediastinal lymph nodes. The combination of biapenem and voriconazole antibiotic treatments was effective. The patient’s respiratory distress was relieved, but there was intermittent coughing. In the following time, the patient developed a fever, and the imaging findings indicated progression of the disease in both lungs. Bronchoalveolar lavage (BAL) identified pathogens of multiple opportunistic infections. The coexistence of lymphomatoid granulomatosis (LYG) was not confirmed in this patient until a second CT-guided biopsy was performed. Ultimately, the patient underwent chemotherapy in time and is currently alive today.

**Conclusions:**

When the patient’s recurrent respiratory symptoms and imaging findings do not coincide, secondary tumors should be considered in addition to infection as a diagnosis. In these cases, multiple pathological tissue biopsies should be performed.

## Introduction

Chronic myeloid leukemia (CML) is a clonal hematopoietic stem cell disorder, characterized by the *BCR-ABL1* fusion gene leading to an aberrant chimeric tyrosine kinase (TK). TK inhibitors (TKIs) can help improve the disease-free survival time in patients with CML. However, the risk of secondary malignancies due to increased survival time of these patients is increased. The cumulative incidence of simultaneous occurrence of CML and other secondary cancers in a singular case has been reported to be less than 5% (1). These mainly comprise cancers of the male genital system and digestive system (2). However, to our knowledge, the diagnosis of primary pulmonary lymphoma (PPL) as a secondary neoplasm during therapy for CML in chronic phase (CML-CP) is extremely rare.

PPL is an uncommon neoplasm that represents 3–4% of all extranodal lymphomas, less than 1% of all lymphomas, and less than 0.5% of all primary pulmonary tumors (3). The rare and nonspecific clinical features of PPL often cause delay and neglect in diagnosis, which negatively affect treatment. A previous study reported a patient who presented with pneumonia-like imaging findings, with a delay in diagnosis of up to 11 years (4). It is common for hematological diseases to be complicated by opportunistic infections, with lung infections being the most common (5). In patients with hematological malignancies, a pulmonary infection may overshadow primary pulmonary lymphoma, posing a challenge for clinicians to diagnose and treat (6). Early identification of the second tumor is particularly important in case of co-infection, to improve outcomes with better treatment options. Here, we present an extremely rare case of simultaneous occurrence of PPL and opportunistic infections in a patient with CML, after 16 months of the primary diagnosis.

## Case description

A 55-year-old woman with no past medical history presented with three weeks of hemorrhage of the oral mucosa at the Department of Hematology in August 2019. There were no complaints of fever, cough, fatigue, or ostalgia. Vital signs revealed a temperature of 36.5°C, heart rate of 80 beats/min, respiratory rate of 20 breaths/min, and blood pressure of 125/81 mmHg. The physical examination revealed unremarkable findings. Laboratory test results revealed a white blood cell count of 88.13×10^9^/L (normal range, 3.5–9.5×10^9^/L) with 67% neutrophils, 2% lymphocytes, 5% basophils, 1% eosinophils, 1% monocytes, 14% myelocytes, 10% metamyelocytes, with an erythrocyte count of 4.18×10^12^/L (normal range, 3.8–5.1×10^12^/L) and a platelet count of 640×10^9^/L (normal range, 125–350×10^9^/L). The lactate dehydrogenase level was 647 U/L (normal range, 135–214 U/L), while erythrocyte sedimentation rate, procalcitonin, liver, and renal function tests were normal. The abdominal ultrasound showed an enlarged spleen. Bone marrow examination showed markedly proliferated granulocytes. The quantitative reverse transcription polymerase chain reaction (RT-PCR) test for *BCR/ABL p210* was positive at the time of diagnosis. Chromosomal analysis revealed 46, XX, t (9,22) (q34; q11). The patient was diagnosed with CML-CP and was started on imatinib (400 mg/daily).

**Figure 1:**
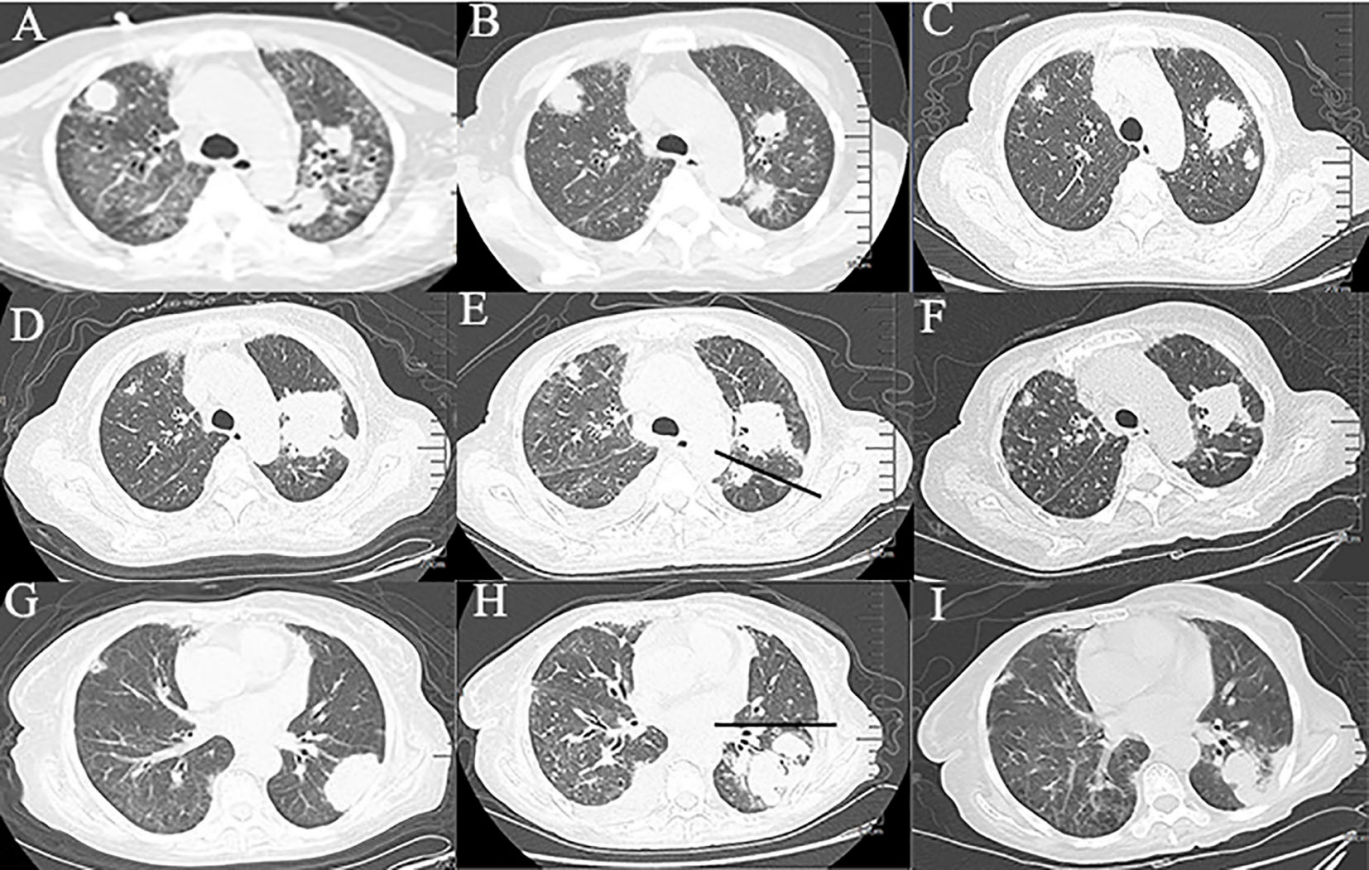
Chest computed tomography at different time periods. **(A‒C)** Changes of ground-glass opacities, patchy shadows, and multiple nodules in both lungs within 50 days. **(D, E)** Changes of masses in the upper lobe of the left lung within 20 days. (The straight line indicates the left interlobar fissure). **(G, H)** Changes of the masses in the lower lobe of the left lung within 20 days. (The straight line indicates the interlobar fissure). **(F, I)** Imaging findings after 2 courses of chemotherapy.

Due to sudden respiratory distress, she was admitted again 11 months later. Her temperature was 36.5°C, heart rate was 137 beats/min, respiratory rate was 34 breaths/min, and blood pressure was 89/64 mmHg. Her cough started 2 months before admission. A physical examination revealed moist rales at the base of the lung. Sputum culture revealed *Stenotrophomonas maltophilia*, filamentous fungi, and traces of *Aspergillus fumigatus*. Routine blood tests showed a white blood cell count of 6.78 ×10^9^/L, an erythrocyte count of 4.00×10^12^/L, and a platelet count of 269×10^9^/L. A peripheral blood smear revealed no abnormal cells. Procalcitonin was 1.035 ng/mL (normal range, 0–0.05 ng/mL). The serum IgM test for toxoplasma, rubella virus, cytomegalovirus, herpes simplex virus and parvovirus B19 (TORCH) was negative. Influenza, mycoplasma pneumoniae, T cell spot test, Epstein–Barr virus (EBV), cytomegalovirus plasma 1-3-β-d glucan test, and plasma galactomannan tests were also negative. An electrocardiogram showed sinus tachycardia. There were no abnormalities on the cardiac ultrasound. Chest computed tomography (CT) revealed ground-glass opacities, patchy shadows, and multiple nodules in both lungs and enlarged mediastinal lymph nodes (**Figure 1A**). She was diagnosed with septic shock and was treated with biapenem and voriconazole. The combination of antibiotic treatments was effective. The patient’s respiratory distress was relieved; however, intermittent coughing remained. Another chest CT showed decreased patchy shadows and multiple nodules in both lungs (**Figures 1B, C**).

She was readmitted 3 months later due to a fever (maximum of 38.5℃). A CT scan showed an increase of the masses in the left lung compared with previous findings (**Figures 1D, G**). CT-guided percutaneous lung biopsy was conducted on October 28, 2020. The pathological examination showed extensive necrosis of the examined tissue. Periodic acid-silver methenamine staining was negative. Simultaneously, she underwent flexible fiberoptic bronchoscopy, which showed thick and yellow secretions. BAL fluid was sent for next-generation sequencing (NGS), which revealed EBV and *Actinomyces odontolyticus*. The patient received penicillin sodium and acyclovir for anti-infective treatment. The patient’s symptoms improved, and she was discharged home from the hospital.

**Figure 2:**
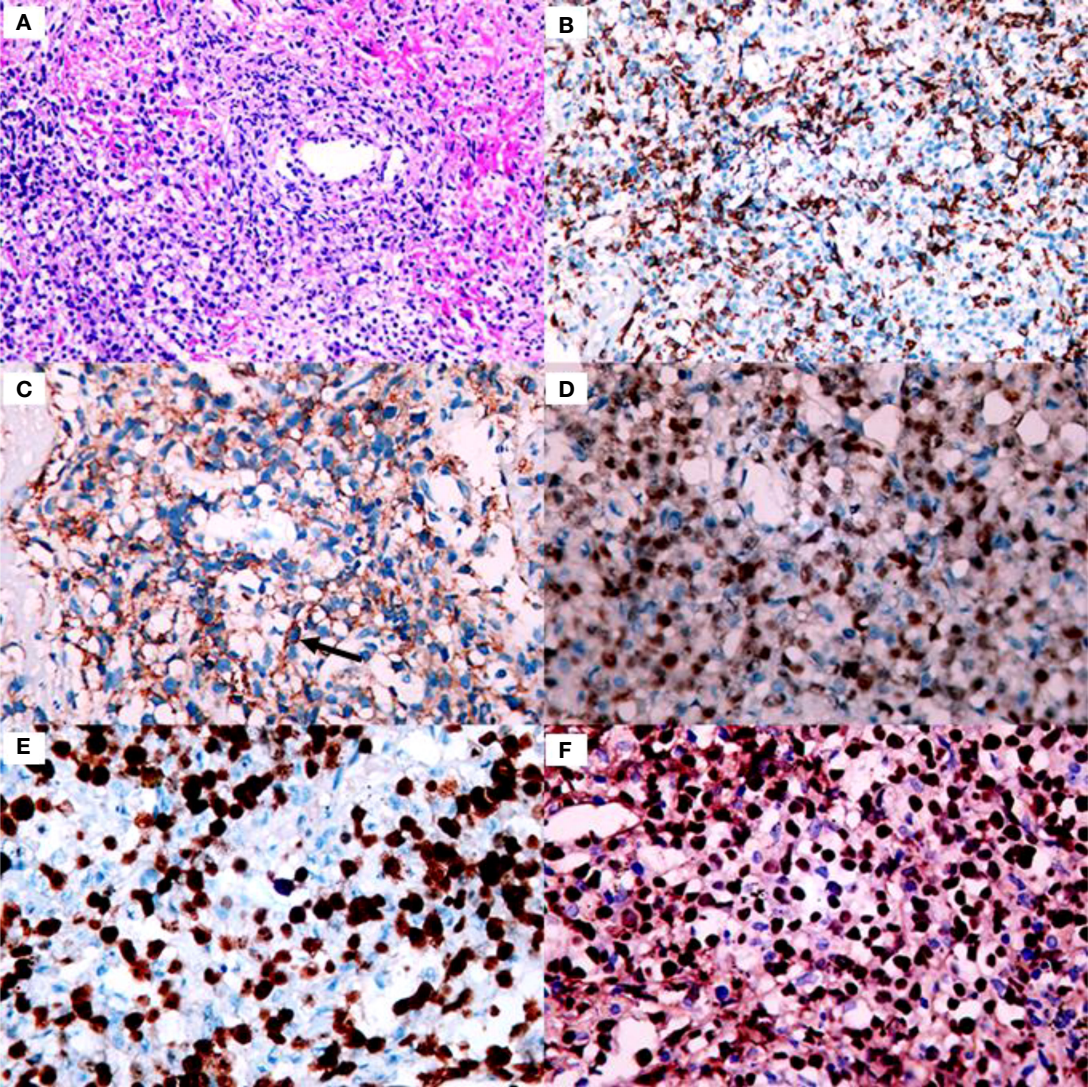
Histology and immunohistochemistry of the core biopsy from the lung is consistent with lymphomatoid granulomatosis grade 3. **(A)** Proliferation and vascular infiltration of polymorphic lymphoid cells (×200 magnification). **(B)** Admixed T cells were positive for CD3 (×200 magnification). **(C)** Scattered CD20 positive large B-cells are detected-with arrows on the picture (×400 magnification). **(D)** The large cells are strongly stained with Pax5 (×400 magnification). **(E)** The large cells are positive for Ki-67 (×400 magnification). **(F)** The number of EBER positive cells is above 50/HPF consistent with garde 3 lymphomatoid granulomatosis (×400 magnification).

**Figure 3:**
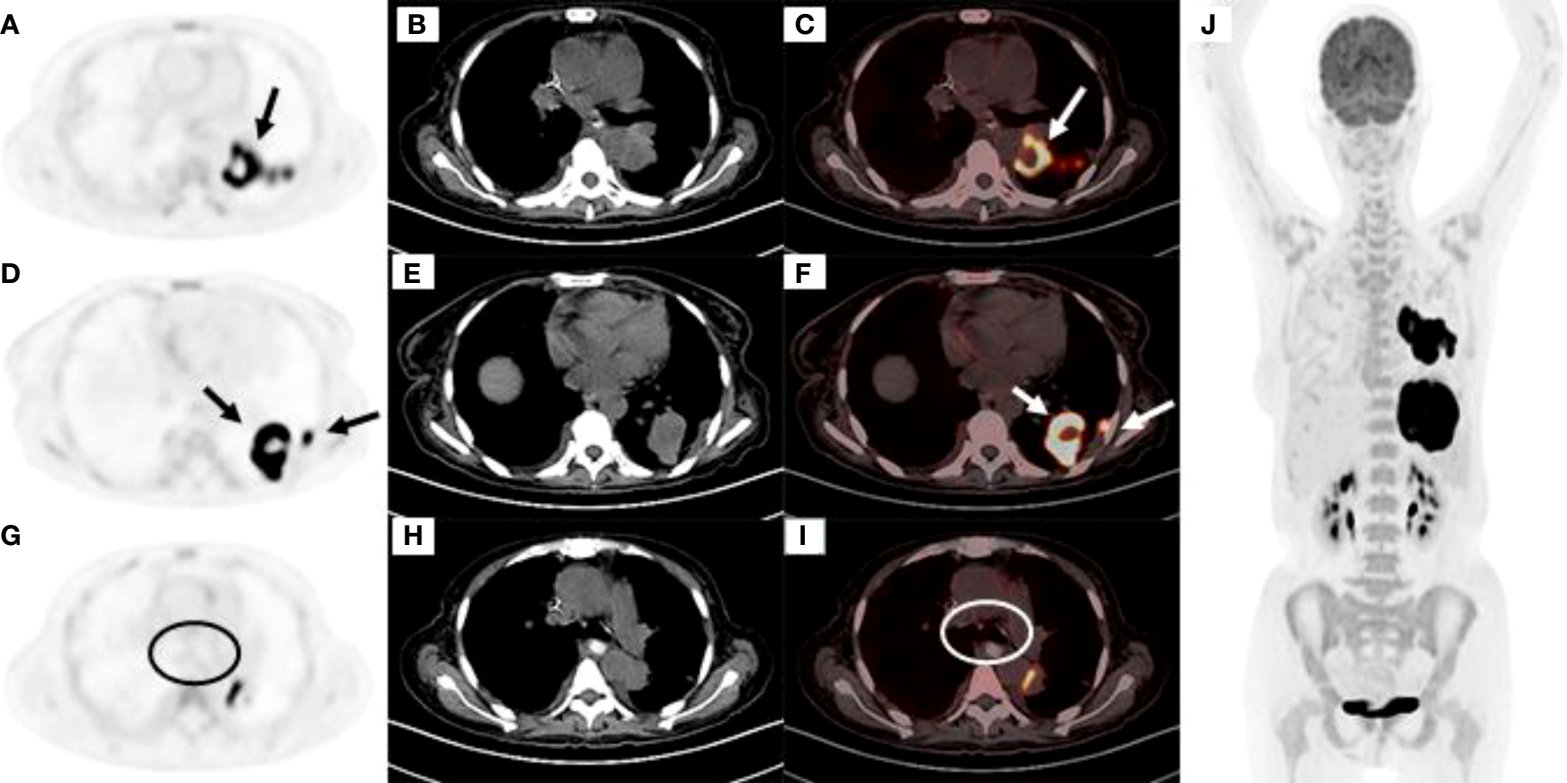
**(A‒C)** High FDG uptake at the dorsal segment of the left lower lobe **(A)** PET image, **(B)** CT mediastinal window, **(C)** PET and CT fusion images. **(D‒F)** High FDG uptake at the posterior basal segment and lateral basal segments of the left lower lobe **(D)** PET image, **(E)** CT mediastinal window, **(F)** PET and CT fusion images. **(G‒I)** No FDG uptake was observed in the mediastinal lymph nodes as shown within the circle **(G)** PET image, **(H)** CT mediastinal window, **(I)** PET and CT fusion images. **(J)** Except for the left lung tumor, there was no obviously high metabolism in other parts of the body.

One month later, the patient presented with worsening cough, white sputum, and intermittent fever (maximum of 38.5°C), and was readmitted. CT revealed a reduction in the size of the masses in the upper lobe of the left lung **(** **Figure 1E** **)** and an increase in the size of the masses in the lower lobe of the left lung compared with the previous scan (**Figure 1H**). Therefore, a second bronchoscopy and bronchoalveolar lavage were performed. Bronchoscopy revealed a white substance in the entrance of the dorsal segment of the left lower lobe. The BAL fluid was sent for NGS, which revealed EBV and *Enterobacter cloacae complex Hoffmann cluster III*. The patient underwent a second CT-guided percutaneous lung biopsy. Histopathological examination of the biopsied lung tissue showed proliferation and vascular infiltration of polymorphic lymphoid cells (**Figure 2**). Immunohistochemical staining showed that the tumor cells were positive for Pax5, CD20, Bcl-2, EBER and (60%) Ki-67 and negative for Bcl-6, c-MYC, CD10, TdT, CD34, CD117, and MPO. MUM-1 staining was positive (20%); admixed T cells were positive for CD3 and CD7; tuberculosis-polymerase chain reaction was negative; and staining of mycobacteria and fungi were negative. Positron emission tomography/computed tomography (PET/CT) revealed an 18 F-fludeoxyglucose (FDG) increased uptake in the dorsal segment, posterior basal segment, and lateral basal segment of the left lower lobe (**Figure 3**). FDG uptake was not detected in the mediastinal lymph nodes, thus excluding secondary lung infiltration of nodal/mediastinal DLBCL (**Figures 3G** **–I**). Bone marrow aspiration did not show involvement of lymphoma cells. *BCR-ABL* (RT-PCR) was 0.0%. Furthermore, laboratory results showed an EBV DNA load of 2.07×10^4^ IU/mL (normal range, <5000 IU/mL) and lactate dehydrogenase level of 348 U/L. The patient was diagnosed with lymphomatoid granulomatosis (grade 3). She stopped imatinib treatment and instead received R-CHOP (rituximab, cyclophosphamide, doxorubicin, vincristine, and prednisone) therapy. Her clinical manifestations improved, and chest CT scan revealed a reduction of the multiple nodules and masses in both lungs after 2 courses of chemotherapy (**Figures 1F, I**). Moreover, five months following imatinib withdrawal, flumatinib was then administered as a treatment for CML. The hematological remission of CML was maintained, and *BCR-ABL* transcript level remained undetectable by RT-PCR. As of August 16, 2022, the patient was still alive.

## Discussion

We experienced a case of a patient who presented with hemorrhage of the oral mucosa and was diagnosed with CML and treated with imatinib. After 11 months, she developed respiratory distress. The initial diagnosis of the patient was a pulmonary infection. After multiple antibiotic treatments, the patient’s pulmonary symptoms and imaging manifestations improved. However, the patient developed fever and the imaging findings continued to progress in both lungs. BAL showed pathogens of multiple opportunistic infections. A second CT-guided biopsy was performed to establish the coexistence of PPL. Finally, the patient underwent chemotherapy in time and is currently alive.

With the success of TKI therapy, the prognosis of patients with CML has remarkably improved. However, secondary therapy-related malignancies may be an associated concern. Sasaki et al. demonstrated that compared with the general population, 13,276 patients with newly diagnosed CML had an increased incidence of a second malignancy (2). Pathogenic theories of these relations remain unclear. Lymphomatoid granulomatosis (LYG) is an EBV-driven B-cell lymphoproliferative disease and is often associated with immunosuppression or immunodeficiency states (7). The main site of involvement is the lungs. Salmons et al. (8). and Yazdi et al. (9). have presented case reports describing LYG following imatinib administration. Both cases were secondary to gastrointestinal stromal tumors. It has been shown that imatinib affects the function of T-lymphocytes and peripheral blood progenitor cells (10, 11). Whether imatinib-induced immunosuppression further promotes LYG development should be considered (8). However, some studies suggest that long-term TKI therapy for patients does not increase the incidence of secondary cancers (1, 12). Pina-Oviedo et al. (13) described a patient with chronic myeloid leukemia, diagnosed with LYG, after 24 years of treatment with interferon and cytarabine. Cytarabine was discontinued and imatinib was used to treat CML. Surprisingly, the patient underwent spontaneous remission of LYG. Therefore, a fear of the possibility of a second tumor secondary to CML should not be a reason for discontinuing TKI therapy. The etiology of coexistence of two haematopoietic malignancies may involve tumor-associated immune deficiency, side effects of therapy, host genetic predisposition, environmental exposures or a combination of these factors (8). Vigilant regular follow-ups are needed in the diagnosis and treatment of patients with CML (14).

PPL is infrequent and defined as a clonal lymphoproliferative disorder that affects one or both lungs (parenchyma and/or bronchi) without extra-pulmonary involvement at the time of diagnosis or over the following three months (15). PPL is rarer than secondary pulmonary lymphoma. The median age for PPL diagnosis is 60 years, and it is more common among woman (15). Mucosa-associated lymphoid tissue lymphoma is the most frequent subtype, accounting for 60% to 80% of all the PPLs, followed by diffuse large B-cell lymphoma (10-25%). Others rare types of PPLs include LYG, mantle B-cell lymphoma and follicular lymphoma (16). About 36% of cases are asymptomatic at the time of diagnosis (17). There are no specific clinical symptoms of PPL. Common symptoms include cough, fever, bloody sputum, dyspnea, and chest pain (18). In previous reports, the imaging findings of PPL were consolidations, ground-glass opacities, and single/multiple nodules, with perilymphatic and/or bronchovascular spread (19). As the clinical manifestations are atypical, most patients are initially misdiagnosed with pulmonary tuberculosis, pneumonia, fungal infections, or lung cancer.

The lung is a common site of opportunistic infections. The risk for an opportunistic infection is influenced by the exposure, host defenses, and interactions between the intrinsic virulence properties of microorganisms and the immune system (20). When pulmonary opportunistic infections occur in patients with hematologic malignancies, they often deteriorate rapidly and evolve to respiratory failure. Several reports have suggested that imatinib may damage the immune system, increasing the risk of infections such as varicella, herpes zoster, and hepatitis B (21). The possibility of infection was considered, and based on the sputum culture and BAL, we identified pathogens of multiple opportunistic infections, including *Stenotrophomonas maltophilia*, filamentous fungi, *Aspergillus fumigatus*, EBV, *Actinomyces odontolyticus*, and *Enterobacter cloacae complex Hoffmann cluster III*. After anti-infective treatment, the patient’s clinical manifestations were significantly improved. Afterward, the patient’s symptoms and imaging findings worsened, which prompted an alternative diagnosis. There are also some cases of co-diagnosed lymphoma and infections, such as invasive pulmonary aspergillosis (6), tuberculosis (22), and *Aspergillus fumigatus* (23). Regardless of the etiology, timely diagnosis and treatment are crucial. After two lung biopsies, we finally established the diagnosis of LYG. Lymphoma may be missed and misdiagnosed when a hematological tumor and infection overlap in the same site.

Our case is the first report of the simultaneous occurrence of PPL and pulmonary infection in a patient with CML. To provide some references for early detection and diagnosis, we offer the following suggestions: First, in the presence of pulmonary symptoms, such as cough, in patients with pre-existing hematologic disorders, the presence of a second neoplasm should be considered in addition to pulmonary infection. Second, when a CT scan shows pneumonia-like imaging findings, it is difficult to differentiate PPL from pneumonia. In our case, after anti-infective treatment, the patient’s symptoms, signs, and imaging findings improved, causing misinterpretation of the findings as recovery. When the patient’s recurrent respiratory symptoms and imaging do not coincide, repeated actively pathological tissue biopsies should be conducted. At last, the accurate diagnosis of PPL requires histopathological examination. The common methods include CT-guided percutaneous lung biopsy, an open thoracotomy or a video-assisted thoracoscopic lung biopsy, and flexible fiberoptic bronchoscopy.

In conclusion, when CML is diagnosed, the presence of lung lesions may lead to consideration of a secondary infection, causing clinicians to overlook other possible diagnoses. Diagnosing concurrent PPL and pulmonary infection is challenging. When the patient’s recurrent respiratory symptoms and imaging do not match, alternative diagnoses should be considered. In such cases, repeated pathological tissue biopsies should be performed.

## Data availability statement

The original contributions presented in the study are included in the article/supplementary material. Further inquiries can be directed to the corresponding authors.

## Ethics statement

The studies involving human participants were reviewed and approved by the Shandong Provincial Qianfoshan Hospital. The patients/participants provided their written informed consent to participate in this study. Written informed consent was obtained from the individual(s) for the publication of any potentially identifiable images or data included in this article.

## Author contributions

YB and SF searched relevant references and drafted the manuscript. JS collected part of the data. QL and YW originated the work, made comments, and revised the manuscript. All authors contributed to the article and approved the submitted version.

## Acknowledgments

The authors thank the patient for allowing us to understand her condition and write this case report.

## Conflict of interest

The authors declare that the research was conducted in the absence of any commercial or financial relationships that could be construed as a potential conflict of interest.

## Publisher’s note

All claims expressed in this article are solely those of the authors and do not necessarily represent those of their affiliated organizations, or those of the publisher, the editors and the reviewers. Any product that may be evaluated in this article, or claim that may be made by its manufacturer, is not guaranteed or endorsed by the publisher.

## Abbreviations

PPL, primary pulmonary lymphoma; CML, chronic myeloid leukemia; CML-CP, CML in chronic phase; CT, computed tomography; BAL, bronchoalveolar lavage; LYG, lymphomatoid granulomatosis; TK, chimeric tyrosine kinase; TKIs, TK inhibitors; NGS, next-generation sequencing; RT-PCR, quantitative reverse transcription polymerase chain reaction; EBV, Epstein–Barr virus; PET/CT, positron emission tomography/computed tomography; FDG, fludeoxyglucose.
